# High plasma levels of parathyroid hormone (PTH) are associated with an increased cardiovascular risk among HIV-infected subjects

**DOI:** 10.1186/1758-2652-13-S4-P233

**Published:** 2010-11-08

**Authors:** V Spagnuolo, L Galli, S Salpietro, C Vinci, F Cossarini, G Travi, A Rubinacci, G Mignogna, M Cernuschi, S Bossolasco, A Lazzarin, A Castagna

**Affiliations:** 1 San Raffaele Scientific Institute, Infectious Diseases Department, via Stamira d'Ancona 20, Milan, Italy; 2 San Raffaele Scientific Institute, Bone Metabolism Unit, via Olgettina 60, Milan, Italy

## Background

Elevated PTH plasma levels are associated to an increased cardiovascular risk (CVR) possibly due to a PTH effect on vascular calcification, myocardial impairment and hypertension.

## Purpose of the study

To assess the association between CVR and PTH levels in HIV-infected patients (pts).

## Methods

HIV-infected subjects followed at the Infectious Disease Department of IRCCS San Raffaele, with at least one PTH plasma value and a contemporary CVR determination. CVR was calculated according to Framingham 10-years equation (Anderson et al.) Cochran-Armitage test for trend and Spearman correlation were calculated at univariate analysis. Logistic regression was applied at multivariable analysis.

## Results

Up to date 280 pts met the inclusion criteria; median (IQR) age was 48.4 (44.4-55.9) years, 205 (73.2%) male, 14.9 (9.3-20.4) years after HIV-infection, 104 (37.1%) smokers, 22 (7.9%) with a previous diagnosis of diabetes, PTH plasma level was 62.7 (49.5-81.5)pg/ml [82 (33%) pts > upper normal limit], vitamin D (25-OH D3) level was 22.5(14.8-31.0) ng/ml, systolic pressure was 120(110-135) mmHg, total cholesterol 192 (169-220) mg/dL, creatinine 0.83 (0.71-0.98) mg/dL, current CD4 cell count was 549 (391-740) cells/mm3 and HIV-RNA< 50cp/ml in 235(83.9%) subjects. Overall CVR was 8 (4.3-13.8)% at 10 years. Eighty-four(30.0%) pts presented a CVR below 5%, 93(33.2%) between 5 and 10%, 46(16.4%) between 10 and 15%, 28(10.0%) between 15 and 20% and 29(10.6%) above 20% risk. PTH levels differed among classes of CVR [CVR <5%, 5-10%, 10-15%, 15-20, >20% had median PTH levels of 57.3, 63.6, 67.0, 73.3 and 65.3 pg/ml, respectively, (p=0.032)]. CVR was positively correlated with PTH (r=0.148, p=0.0134), creatinine (r=0.316, p<0.0001), triglycerides (r=0.300, p<0.0001) but not with vitamin D level (r= -0.102; p=0.123). After adjusting for PTH plasma levels, years of antiretroviral therapy (ART), years of HIV-infection, nadir and current CD4 cell count, detectable HIV-RNA, HIV transmission risk factor, triglycerides and creatinine levels, a CVR>10% was predicted by increasing PTH levels (OR=1.353 per 20-pg/mL increase, 95%CI: 1.074-1.802, p=0.028)as well as by increasing creatinine levels (OR=1.442 per 0.1-mg/dL increase, 95%CI:1.201-1.774, p=0.0002).

## Conclusions

Among HIV-infected patients elevated PTH levels were related to increased CVR and independently predicted a 10-years CVR above 10%. The interplay between bone metabolism and CVR needs to be further investigated.

